# Outcomes following surgery in subgroups of comatose and very elderly patients with chronic subdural hematoma

**DOI:** 10.1007/s10143-018-0979-4

**Published:** 2018-04-21

**Authors:** Edward Christopher, Michael T. C. Poon, Laurence J. Glancz, Peter J. Hutchinson, Angelos G. Kolias, Paul M. Brennan, on behalf of the British Neurosurgical Trainee Research Collaborative (BNTRC), F. T. Afshari, A. I. Ahmed, S. Alli, R. Al-Mahfoudh, J. Bal, A. Belli, A. Borg, D. Bulters, N. Carleton-Bland, A. Chari, D. Coope, I. C. Coulter, C. J. Cowie, G. Critchley, S. Dambatta, D. D’Aquino, B. Dhamija, G. Dobson, M. D. Fam, W. P. Gray, B. A. Gregson, P. J. Grover, J. Halliday, A. Hamdan, C. S. Hill, A. A. B. Jamjoom, A. J. Joannides, T. L. Jones, S. M. Joshi, A. Kailaya-Vasan, V. Karavasili, S. A. Khan, A. T. King, A. Kuenzel, L. J. Livermore, W. Lo, H. J. Marcus, J. Martin, S. Matloob, P. Mitchell, D. Mowle, H. Narayanamurthy, R. J. Nelson, D. Ngoga, I. Noorani, G. O’Reilly, H. Othman, K. Owusu-Agyemang, K. S. Manjunath Prasad, P. Plaha, J. Pollock, K. S. Prasad, R. Price, C. Pringle, A. Ray, J. Reaper, W. Scotton, J. Shapey, N. Simms, S. Smith, P. Statham, L. Steele, J. St George, M. G. Stovell, A. Tarnaris, M. Teo, S. Thomson, L. Thorne, M. Vintu, P. Whitfield, M. Wilson, M. Wilby, J. Woodfield, M. Zaben

**Affiliations:** 1 College of Medicine and Veterinary Medicine, University of Edinburgh, Edinburgh, UK; 2 Translational Neurosurgery, Centre for Clinical Brain Sciences, University of Edinburgh and Western General Hospital, Edinburgh, UK; 3 Department of Neurosurgery, Queen’s Medical Centre, Nottingham, UK; 4 Division of Neurosurgery, Department of Clinical Neurosciences, University of Cambridge and Addenbrooke’s Hospital, Cambridge, UK; 5 Surgery Theme, Cambridge Clinical Trials Unit, Cambridge Biomedical Campus, Cambridge, UK; 6 Department of Clinical Neurosciences, Western General Hospital, Edinburgh, UK

**Keywords:** Chronic subdural hematoma, Very elderly, Comatose, Outcome, Surgery

## Abstract

Increasing age and lower pre-operative Glasgow coma score (GCS) are associated with worse outcome after surgery for chronic subdural haematoma (CSDH). Only few studies have quantified outcomes specific to the very elderly or comatose patients. We aim to examine surgical outcomes in these patient groups. We analysed data from a prospective multicentre cohort study, assessing the risk of recurrence, death, and unfavourable functional outcome of very elderly (≥ 90 years) patients and comatose (pre-operative GCS ≤ 8) patients following surgical treatment of CSDH. Seven hundred eighty-five patients were included in the study. Thirty-two (4.1%) patients had pre-operative GCS ≤ 8 and 70 (8.9%) patients were aged ≥ 90 years. A higher proportion of comatose patients had an unfavourable functional outcome (38.7 vs 21.7%; *p* = 0.03), although similar proportion of comatose (64.5%) and non-comatose patients (61.8%) functionally improved after surgery (*p* = 0.96). Compared to patients aged < 90 years, a higher proportion of patients aged ≥ 90 years had unfavourable functional outcome (41.2 vs 20.5%; *p* < 0.01), although approximately half had functional improvement following surgery. Mortality risk was higher in both comatose (6.3 vs 1.9%; *p* = 0.05) and very elderly (8.8 vs 1.1%; *p* < 0.01) groups. There was a trend towards a higher recurrence risk in the comatose group (19.4 vs 9.5%; *p* = 0.07). Surgery can still provide considerable benefit to very elderly and comatose patients despite their higher risk of morbidity and mortality. Further research would be needed to better identify those most likely to benefit from surgery in these groups.

**Electronic supplementary material:**

The online version of this article (10.1007/s10143-018-0979-4) contains supplementary material, which is available to authorized users.

## Introduction

Chronic subdural haematoma (CSDH) is a common neurosurgical condition whose incidence and prevalence increase with age [1–4]. Surgical drainage is the gold standard treatment for the management of symptomatic CSDH [5, 6]. Longitudinal studies have consistently demonstrated that age and pre-operative Glasgow coma score (GCS) are associated with worse surgical outcomes [7, 8]. However, whilst older patients and comatose patients are in a poorer prognostic group, the outcomes specific to these patients are not well studied. Quantifying these would inform clinical judgement.

With an ageing population, determining the optimal management of CSDH in older people is of increasing importance. Whilst the median age of patients admitted to neurosurgical units (NSUs) for surgery is 77 [9], no consensus currently exists in the management of very elderly patients with CSDH (≥ 90 years old). Surgery may hence be delayed or refused, because of the increased risk of perioperative morbidity and mortality and the perception of an expected likelihood of uniformly poor outcomes [10]. Paradoxically, this vulnerable group may benefit most from urgent surgery [10]. Uncertainties also remain as to whether comatose patients should undergo surgery at all.

In this study, we analysed data from a prospective multicentre cohort study to assess the risk of recurrence, risk of death, and functional outcome of patients aged ≥ 90 and comatose patients following surgical drainage of CSDH, to inform decision-making in this population.

## Methods

The detailed study protocol for the prospective multicentre study has been published previously [9, 11]. Briefly, this was a prospective multicentre cohort study in the UK and Ireland that aimed to describe the perioperative clinical characteristics and short-term outcomes of patients with CSDH. There were 26 NSUs recruiting patients during the study period between May 2013 and January 2014. Patients were eligible for the study if they were (1) ≥ 16 years of age; (2) diagnosed with a primary or recurrent CSDH confirmed on neuroimaging; and (3) referred to a NSU. CSDH was radiologically defined as a primarily hypodense, isodense, or mixed-density subdural collection. Patients were excluded from the study if other pathologies (e.g. subdural empyema, vascular malformations) were identified during operation or during subsequent management. There were no pre-specified criteria for offering surgery to patients with CSDH; the decision was made by the respective consultant neurosurgeon on duty. Patient data recorded included demographics (age and gender), baseline characteristics (comorbidities, history of head injury, antithrombotic drug use, pre-operative clinical status), and perioperative management (platelet transfusion, vitamin K use, operation lateralization, operation type, drain insertion, and post-operative bed rest instructions). Data was stored using a secure online database that complies with the Department of Health Information Governance policies. Each NSU had received local clinical governance approval.

### Outcome measures

For this current study, patients with a pre-operative GCS of ≤ 8 were considered as comatose. Patients aged ≥ 90 years were considered as very elderly. Data from these patients was extracted from the master database for analysis. Outcome measures included recurrence, functional status at discharge, and mortality. We defined recurrence as radiologically confirmed symptomatic recurrence requiring re-operation within 60 days of index admission. There were two functional status outcome measures. First was the early absolute functional status as measured using the modified Rankin Scale (mRS) on discharge from NSU. A score of 0–3 had been pre-specified as favourable, whilst 4–6 as unfavourable. Second is a relative functional improvement comparing the mRS on discharge with the pre-operative mRS. This measure was defined post hoc and was not included in the original protocol [9, 11]. Mortality was defined as death due to any cause that occurred during admission in the NSU.

### Statistical methods

Baseline characteristics were compared using parametric and non-parametric tests as appropriate. We used chi-square or Fisher’s exact test to compare outcome measures between groups. Wilcoxon paired test was used to compare pre- and post-operative mRS scores within a group. The number of available outcomes precludes multivariate analyses. All statistical analyses were performed using Stata version 13.0 (StataCrop).

## Results

Data was collected for 1205 patients with CSDH referred to 26 NSUs. Seven hundred eighty-five patients (65.1%) were subsequently accepted for NSU admission upon referral and were included in our study. Of these 785 patients, 32 patients (4.1%) had GCS ≤ 8 and 70 patients (8.9%) were ≥ 90 years of age.

### Patients with GCS ≤ 8 undergoing surgery

#### Baseline characteristics of patients with pre-operative GCS ≤ 8

There were 32 patients with pre-operative GCS ≤ 8. The baseline characteristics of these patients are presented in Online Resource 1. Characteristics of comatose patients were similar to non-comatose patients. Age, gender, comorbidities, and peri-operative management were similar between the two groups. We were unable to compare the baseline characteristics of comatose patients who were transferred with those who stayed in the referring hospital, as detailed data was not collected for patients not transferred to NSU.

#### Outcomes of patients with pre-operative GCS ≤ 8

Table 1 summarises outcomes in comatose patients. Two (6.3%) deaths occurred in the comatose group compared with the 14 (1.9%) deaths in the non-comatose group (*p* = 0.05). There were six (19.4%) recurrences occurring within 60 days in the comatose group; there was a trend of higher recurrence risk in the comatose group compared with the risk in the non-comatose group (9.5%) (*p* = 0.07). The proportion of patients with unfavourable functional outcome (discharge mRS 4–6) in the comatose group was higher than that in the non-comatose group (*p* = 0.03). However, comparing the initial mRS with the discharge mRS using paired analyses, there was a significant improvement in functional status in both comatose and non-comatose groups following surgery (*p* < 0.01). A similar proportion of comatose and non-comatose patients functionally improved after surgery (*p* = 0.96).

**Table 1:** Summary table of outcome measures in comatose and non-comatose groups. Patients with incomplete data were excluded from analyses

	Number of events *n*/*N* (%)		*p* value
	Comatose group	Non-comatose group	
Recurrence within 60 days	6/31 (19.4)	70/733 (9.5)	0.07
Discharge mRS 4–6	12/31 (38.7)	159/733 (21.7)	0.03
No functional improvement	11/31 (35.5)	280/733 (38.2)	0.96
Death	2/32 (6.3)	14/753 (1.9)	0.05

### Patients aged ≥ 90 years undergoing surgery

#### Baseline characteristics of patients aged ≥ 90 years

The baseline characteristics of the 70 patients aged ≥ 90 years are presented in Online Resource 2. Characteristics of patients aged ≥ 90 years were similar to patients aged < 90 years except the older group having less male predominance (*p* = 0.02), higher proportion of patients reporting a history of head injury within the preceding 3 months (*p* < 0.01), lower pre-operative functional status (*p* < 0.01), lower pre-operative GCS (*p* = 0.04), and more likely to have undergone an operation under local anaesthesia (*p* < 0.01). Comorbidities and peri-operative management were otherwise similar between the two groups. We were unable to compare the baseline characteristics of patients aged ≥ 90 years who were transferred with those who stayed in the referring hospital, as detailed data were not collected for patients not transferred to NSU.

#### Outcomes of patients aged ≥ 90 years

Table 2 summarises the outcomes in the older and younger groups of patients. Six (8.8%) deaths occurred in the older group compared with eight (1.1%) in the younger group (*p* < 0.01). There was no difference in the risk of recurrence between the older group (11.8%) and the younger group (9.8%) (*p* = 0.60). In the older group, there were 28 (41.2%) patients with an unfavourable functional outcome at discharge and 35 (51.5%) patients with no functional improvement at discharge. These proportions were significantly higher than in the younger group (*p* < 0.01). However, comparing the initial mRS with the discharge mRS using paired analyses, there was a significant improvement in functional status in both older and younger groups following surgery (*p* < 0.01).

**Table 2:** Summary table of outcome measures in older and younger patients. Patients with incomplete data were excluded from analyses

	Number of events *n*/*N* (%)		*p* value
	Age ≥ 90 years	Age < 90 years	
Recurrence within 60 days	8/68 (11.8)	68/696 (9.8)	0.60
Discharge mRS 4–6	28/68 (41.2)	143/696 (20.5)	< 0.01
No functional improvement	35/68 (51.5)	239/696 (34.3)	< 0.01
Death	6/68 (8.8)	8/696 (1.1)	< 0.01

In our cohort, there were three patients aged ≥ 90 years presenting in a coma. They all had poor functional status pre-operatively. One patient died; the remaining had same or worse functional outcome following surgery.

## Discussion

About a tenth of the patients referred to neurosurgery were ≥ 90 years of age in our study, which is similar to a population-based study [12]. Similar to our findings, previous studies have reported increased mortality following CSDH surgery in patients aged ≥ 90 years [7, 8, 13–15]. We also found that a higher proportion of patients aged ≥ 90 years had unfavourable outcome and about half did not have functional improvement post-operatively. Multiple comorbidities, reduced physiological reserve, worse pre-morbid functional status, and the natural increased risk of death in very elderly people are all factors that are likely to contribute towards this. We also found that increasing age does not appear to be associated with increased recurrence risk, consistent with previous studies [9, 12, 16–20].

In addition to higher mortality, patients aged ≥ 90 years had worse functional outcome compared to younger patients. In fact, in a recent large Japanese population-based study, analysis of a subgroup of 5414 patients aged ≥ 90 found that 56.8% had a poor outcome (mRS 3–6) and under 40% of them were discharged home [12]. Whilst patients aged ≥ 90 had worse functional status pre-operatively, our findings support this observation. Our findings also demonstrate an overall significant improvement in functional status after surgery in the paired analyses. This benefit from surgery was not seen in all very elderly patients. It is also not possible to know from our data whether these functional improvements were sufficient to impact on the patient’s quality of life. Future studies should aim to identify factors that predict which very elderly patients are most likely to make functional benefits. We could not exclude further improvements made by very elderly patients following their discharge from hospital; however, mid-term and long-term data were not collected as part of this study.

Under 3% of all the referred patients in this cohort were in coma (GCS ≤ 8), which is similar to a Scandinavian population-based cohort [17]. Other studies based in Iran [16] and India [7] reported a much higher proportion of comatose patients (10 and 14% respectively), presumably reflecting variations in referral practices. We found that comatose patients had worse functional outcome at discharge and higher mortality risk compared to non-comatose patients. Previous single-institutional retrospective study with a subgroup of 92 surgically treated comatose patients reported 30% death and 35% moderate to severe disability risk [7]. This can be explained by their worse functional baseline on admission, since pre-operative mRS is a predictor of post-operative outcome [9]. We also observed a trend towards higher recurrence risk in comatose patients which did not reach statistical significance.

Nonetheless, in a subset of comatose patients, there may be benefit to surgery. In the paired analyses, we found an overall significant improvement in functional status following surgery in comatose patients. Duration of coma and pupillary reaction could influence surgical outcome in comatose patients [21] and this may explain why surgery was not beneficial in all comatose patients. Duration of coma and pupillary reaction were not collected as part of this study and we were therefore unable to confirm nor refute these relationships. Interestingly, similar proportions of comatose and non-comatose patients functionally improved following surgery. Low GCS is associated with poorer outcomes [7, 8], but should not be used for patient selection alone. The context of the patient’s overall condition is important.

Overall, our findings support the notion that comatose and very elderly patients with CSDH are at a higher risk of morbidity and mortality from surgery. However, advanced age and low GCS do not preclude clinical improvement and so these patients should not be routinely denied surgery. With improved understanding of the pathophysiology of CSDH [22, 23], effective non-surgical therapies may potentially obviate the need for surgery in these vulnerable patient groups. But for now, non-surgical management provides little to no functional improvement [13, 14]. Surgery remains the gold standard treatment for the management of symptomatic CSDH [5, 6].

Although we have demonstrated that surgery can significantly improve outcomes for the very elderly and comatose, patients will vary in their acceptance of the trade-off against surgical risk [24, 25]. The impact on outcomes following surgery reported in recent randomised controlled trials such as DECRA [26] and RESCUEicp [27] have highlighted that patients’ opinion on whether a particular surgical outcome is considered as improvement or not will vary. Our data will assist clinicians in providing patients, their relatives, and their carers with a realistic assessment of the likely benefits of surgery.

Our study has several limitations. There are differences in baseline characteristics in the subgroups of interest and their comparison groups. Analyses adjusting for potential confounders were not possible due to the few outcome events in the small subgroups, which prevented multivariate analyses. This study design did not record the criteria for patient selection and these criteria are likely to be different between NSUs. Because of this, we were unable to comment on or recommend factors to consider when assessing patients in these high-risk groups. Further research would be needed in order to better identify pre-operatively which comatose and very elderly patients are likely to benefit the most from surgery. Our dichotomy of age groups did not lend us to examine the association between increasing age and outcome, though this was not our objective. Although we are able to focus on the high-risk groups of patients aged ≥ 90 years or in a coma, fully adjusted analyses to examine outcomes were not achievable due to paucity of outcome events. We were unable to examine patients aged ≥ 90 years who are comatose due to small patient numbers. Future studies should explore this combined high-risk group. Our study design did not allow longer term following-up of these patients beyond 60 days.

In conclusion, patients aged ≥ 90 years or are comatose pre-operatively are in a high-risk group with increased mortality and morbidity. Surgical management has a role in achieving more favourable outcomes in carefully selected patients. The challenge is to develop a strategy to identify those who will improve with surgery from those who will not.

## Electronic supplementary material

Online Resource 1 (DOC 64 kb)

Online Resource 2 (DOC 63 kb)
