# Supplementary material for: Outcomes following surgery in subgroups of comatose and very elderly patients with chronic subdural hematoma
Source: Neurosurg Rev. 2018 Apr 21;42(2):427–31. doi: 10.1007/s10143-018-0979-4 (PMC6502770; doi:10.1007/s10143-018-0979-4)
Supplement: Supplementary file 2 — (DOC 63 kb) [file 10143_2018_979_MOESM2_ESM.doc]

**Outcomes following surgery in subgroups of comatose and very elderly patients with chronic subdural hematoma**

Edward Christopher^1*^, Michael T C Poon^2*^, Laurence J Glancz^3^, Peter J Hutchinson^4,5^, Angelos G Kolias^4,5^, Paul M Brennan^2^ on behalf of the British Neurosurgical Trainee Research Collaborative (BNTRC)

*^1^College of Medicine and Veterinary Medicine, University of Edinburgh, UK*

*^2^Translational Neurosurgery, Centre for Clinical Brain Sciences, University of Edinburgh and Western General Hospital, Edinburgh, UK*

*^3^Department of Neurosurgery, Queen’s Medical Centre, Nottingham, UK*

*^4^Division of Neurosurgery, Department of Clinical Neurosciences, University of Cambridge and Addenbrooke’s Hospital, Cambridge, UK*

*^5^Surgery Theme, Cambridge Clinical Trials Unit, Cambridge Biomedical Campus, Cambridge, UK*

**These authors contributed equally to this work*

Corresponding author:

Paul Brennan

Department of Clinical Neurosciences

Western General Hospital

Edinburgh

email: [paul.brennan@ed.ac.uk](mailto:paul.brennan@ed.ac.uk)

**Acknowledgments**

Peter Hutchinson is supported by a Research Professorship from the National Institute for Health Research (NIHR), the NIHR Cambridge Biomedical Research Centre, a European Union Seventh Framework Program grant (CENTER-TBI; grant no. 602150), and the Royal College of Surgeons of England. Angelos Kolias is supported by a Clinical Lectureship, School of Clinical Medicine, University of Cambridge.

**Conflicts of interest**

We declare no conflicts of interest.

**Ethical approval**

Ethical approval is not applicable as this study is the analysis of patient subgroups from a previously published study. The Academic Committee of the Society of British Neurological Surgeons (SBNS) approved the protocol of the original study. The original study was supported by the SBNS and formed part of the Neurosurgical National Audit Program.

**British Neurosurgical Trainee Research Collaborative collaborators**

Afshari FT, Ahmed AI, Alli S, Al-Mahfoudh R, Bal J, Belli A, Borg A, Bulters D, Carleton-Bland N, Chari A, Coope D, Coulter IC, Cowie CJ, Critchley G, Dambatta S, D’Aquino D, Dhamija B, Dobson G, Fam MD, Gray WP, Gregson BA, Grover PJ, Halliday J, Hamdan A, Hill CS, Jamjoom AAB, Joannides AJ, Jones TL, Joshi SM, Kailaya-Vasan A, Karavasili V, Khan SA, King AT, Kuenzel A, Livermore LJ, Lo W, Marcus HJ, Martin J, Matloob S, Mitchell P, Mowle D, Narayanamurthy H, Nelson RJ, Ngoga D, Noorani I, O’Reilly G, Othman H, Owusu-Agyemang K, Manjunath Prasad KS, Plaha P, Pollock J, Prasad KS, Price R, Pringle C, Ray A, Reaper J, Scotton W, Shapey J, Simms N, Smith S, Statham P, Steele L, St George J, Stovell MG, Tarnaris A, Teo M, Thomson S, Thorne L, Vintu M, Whitfield P, Wilson M, Wilby M, Woodfield J, Zaben M.

**Online Resource 2. Baseline characteristics of patients aged below and above 90 years**

|  | Age <90 years  (n=715) | Age ≥90 years  (n=70) | P-value |
| --- | --- | --- | --- |
| Median age (IQR) | 76 (67-83) | 92 (91-94) | - |
| Gender |  |  | 0.02 |
| Female | 222 (31.1) | 31 (44.3) |  |
| Male | 493 (68.9) | 39 (55.7) |  |
| Co-morbidities |  |  |  |
| Diabetes | 117 (16.4) | 8 (11.3) | 0.28 |
| Dementia | 74 (10.4) | 12 (17.1) | 0.08 |
| COPD | 40 (5.6) | 3 (4.3) | 0.65 |
| Cerebrovascular event | 114 (15.9) | 13 (18.6) | 0.56 |
| Ischaemic heart disease | 174 (24.3) | 19 (27.1) | 0.60 |
| Arrhythmia | 147 (20.6) | 14 (20.0) | 0.91 |
| Epilepsy | 32 (4.5) | 1 (1.4) | 0.25 |
| CSF shunt | 7 (1.0) | 0 (0) | 0.41 |
| Malignancy | 60 (8.4) | 6 (8.6) | 0.96 |
| Metallic heart valve | 14 (2.0) | 3 (4.3) | 0.21 |
| History of head injury | 441 (61.7) | 54 (77.1) | <0.01 |
| Antithrombotics |  |  | 0.33 |
| No antithrombotics | 403 (56.4) | 46 (65.7) |  |
| Antiplatelet | 151 (21.1) | 12 (17.1) |  |
| Anticoagulant | 131 (18.3) | 9 (12.9) |  |
| Other | 30 (4.2) | 3 (4.3) |  |
| Pre-operative mRS |  |  | <0.01 |
| mRS 0-3 | 428 (59.9) | 29 (41.4) |  |
| mRS 4-5 | 287 (40.1) | 41 (58.6) |  |
| Platelet transfusion | 69 (9.7) | 5 (7.1) | 0.49 |
| Vitamin K | 116 (16.2) | 9 (12.9) | 0.46 |
| Clotting factors | 18 (2.5) | 0 (0) | 0.18 |
| Pre-operative GCS |  |  | 0.04 |
| Median (IQR) | 14 (14-15) | 14 (14-14) |  |
| GCS 3-8 | 29 (4.1) | 3 (4.3) |  |
| GCS 9-12 | 90 (12.6) | 10 (14.3) |  |
| GCS 13-15 | 596 (83.4) | 57 (81.4) |  |
| Operation lateralisation |  |  | 0.33 |
| Unilateral | 551 (77.1) | 49 (70.0) |  |
| Bilateral | 162 (22.7) | 19 (27.1) |  |
| Unknown | 2 (0.3) | 2 (2.9) |  |
| Operation |  |  | 0.96 |
| Burr hole drainage | 635 (88.8) | 62 (88.6) |  |
| Mini-craniotomy | 65 (9.1) | 5 (7.1) |  |
| Others | 13 (1.8) | 1 (1.4) |  |
| Unknown | 2 (0.3) | 2 (2.9) |  |
| Drain inserted* | 591 (82.9) | 62 (91.2) | 0.08 |
| Pre-operative maximal thickness | 24 (18-30) | 26 (19-32) | 0.15 |
| Post-operative bed rest |  |  | 0.77 |
| No restriction | 290 (40.6) | 27 (38.6) |  |
| Instructed | 423 (59.2) | 41 (58.6) |  |
| Unknown | 2 (0.3) | 2 (2.9) |  |
| Anaesthetics* |  |  | <0.01 |
| General | 671 (93.8) | 55 (78.6%) |  |
| Local | 42 (5.9%) | 13 (13.6%) |  |

*4 patients had missing data on drain insertion and anaesthetics
